# Mechanisms of the Gastric Antiulcerogenic Activity of *Anacardium humile* St. Hil on Ethanol-Induced Acute Gastric Mucosal Injury in Rats

**DOI:** 10.3390/molecules15107153

**Published:** 2010-10-15

**Authors:** Anderson Luiz-Ferreira, Ana Cristina Alves de Almeida, Maíra Cola, Victor Barbastefano, Ana Beatriz Albino de Almeida, Leônia Maria Batista, Elisângela Farias-Silva, Cláudia Helena Pellizzon, Clélia Akiko Hiruma-Lima, Lourdes Campaner Santos, Wagner Vilegas, Alba Regina Monteiro Souza Brito

**Affiliations:** 1 Departamento de Anatomia, Biologia Celular, Fisiologia e Biofísica, Instituto de Biologia, Universidade Estadual de Campinas, Campinas, SP, Brazil; E-Mails: aninhadabio@gmail.com (A‑C.A.A.); mcola1@yahoo.com.br (M.C.); vicbio@yahoo.com (V.B.); anabia5@yahoo.com.br (A‑B.A.A.); elisfarias.com@gmail.com (E.F-S); abrito@unicamp.br (A.R.M.S-B); 2 Laboratório de Tecnologia Farmacêutica, Universidade Federal da Paraíba - UFPB, Cx. Postal 5009, 58051-970, João Pessoa, PB, Brazil; E-Mail: leoniab@uol.com.br (L-M.B.); 3 Departamento de Morfologia, Instituto de Biociências, Universidade Estadual Paulista, Botucatu, SP, Brazil; E-Mail: claudia@ibb.unesp.br (C-H.P.); 4 Departamento de Fisiologia, Instituto de Biociências, Universidade Estadual Paulista, Botucatu, SP, Brazil; E-Mail: hiruma@ibb.unesp.br (C.A.H-L.); 5 Departamento de Química Orgânica, Instituto de Química, Universidade Estadual Paulista, Araraquara, SP, Brazil; E-Mails: loursant@iq.unesp.br (L-C.S.); vilegasw@gmail.com (W.V.)

**Keywords:** *Anacardium humile*, antiulcer activity, cytoprotection, medicinal plants

## Abstract

Leaves and bark infusions *Anacardium humile* St. Hil. (Anacardiaceae), known as in Brazil as “cajuzinho do cerrado”, have been used in folk medicine as an alternative treatment for ulcers and gastritis. This study evaluated the gastroprotective activity of an ethyl acetate extract of the leaves of *A. humile* (AcF) and the mechanism involved in this gastroprotection. Pretreatment concentrations (50, 100, 200 mg.kg^−1^) were administered by gavage. Following a 60 min. period, all the rats were orally administered 1 mL of absolute ethanol. One hour after the administration of ethanol, all groups were sacrificed, and the gastric ulcer index was calculated. Prostaglandin PGE_2_ concentration, gastric adherent mucous, and the participation of nitric oxide (NO) and sulfhydryl compounds in the gastroprotection process were also analyzed using the most effective tested dose (50 mg·kg^−1^). A histological study of the glandular stomach for the evaluation of the epithelial damage and mucus content was also performed. AcF significantly reduced the gastric damage produced by ethanol. This effect was statistically significant for the 50 mg·kg^−1^ group compared to control. Also, it significantly increased the PGE_2_ (by 10-fold) and mucous production, while pretreatment with NG-nitro-L-arginine methyl ester (L-NAME) or *N*-ethylmaleimide (NEM) completely abolished the gastroprotection. AcF has a protective effect against ethanol, and this effect, might be due to the augmentation of the protective mechanisms of mucosa.

## Introduction

Numerous natural products derived from plant sources have been evaluated as therapeutics for the treatment of various diseases [1]. Among these diseases, peptic ulcers are a common disorder of the entire gastrointestinal tract that occur mainly in the stomach and the proximal duodenum. Despite great advances in the understanding of the peptic ulcer illness, its etiology has not been completely elucidated. The basic physiopathological concept is that the peptic ulcer results from an imbalance between some endogenous aggressive factor(s) [hydrochloric acid, pepsin, refluxed bile, leukotrienes, reactive oxygen species (ROS)] and cytoprotective factors, which include the function of the mucus-bicarbonate barrier, surface active phospholipids, prostaglandins (PGs), mucosal blood flow, cell renewal and migration, nonenzymatic and enzymatic antioxidants and some growth factors [2,3,4,5]. 

Although recent advances in our understanding have highlighted the multi-factorial pathogenesis of peptic ulcers, secretion of gastric acid is still recognized as a central component of this disease. Therefore, the main therapeutic target is the control of this secretion using antacids, H_2_ receptor blockers (ranitidine, famotidine) or proton pump blockers (omeprazole and lansoprazole) [6]. However, nowadays gastric ulcer therapy faces a major drawback because most of the drugs currently available in the market show limited efficacy against gastric diseases and are often associated with severe side effects [7,8].

In this context, the use of medicinal plants for the prevention and treatment of different pathologies is in continuous expansion all over the world, including the subject of this research [9]. Natural products are gaining space and importance in the pharmaceutical industry as well as inspiring the search for new potential sources of bioactive molecules [10,11].

*Anacardium humile* St. Hil. (Anacardiaceae), popularly known as “cajuzinho do cerrado” or “cajuí” is a shrub (≅ 30 cm tall), with very long roots, small flowers, a greenish calyx and red petals with stripes. The leaves and bark infusions are used in folk medicine as anti-emetics and diuretics, and as treatments for ulcers, gastritis and diarrhea [12,13,14]. In this regard, *Anacardium humile* St. Hil. extracts have shown to possess antiulcerogenic activity [15]. 

The aim of this work was to evaluate the pharmacological role of this species, describing the mechanism involved in the gastroprotective action of the ethyl acetate extract (AcF) of leaves of *Anacardium humile* St. Hil. in order to justify whether the traditional use of this medicinal species for ulcer, gastritis or diarrhea is justified. 

## Results and Discussion

Historically, natural products have provided an endless source of alternative drugs to medicine. Plant-derived products have dominated the human pharmacopoeia for thousands of years almost unchallenged [16]. In Brazil, a large number of herbal extracts are used in folk medicine to treat various types of digestive disorders [17], including the specie *Anacardium humile*. 

**Figure 1:**
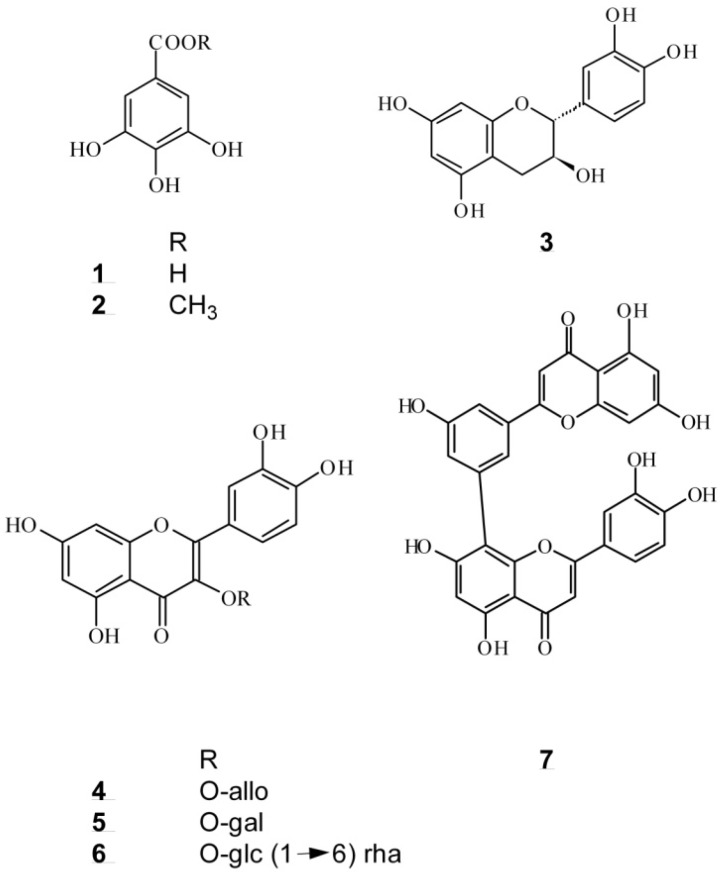
Compounds found in the leaves of *Anacardium humile* St. Hil.

**Figure 2:**
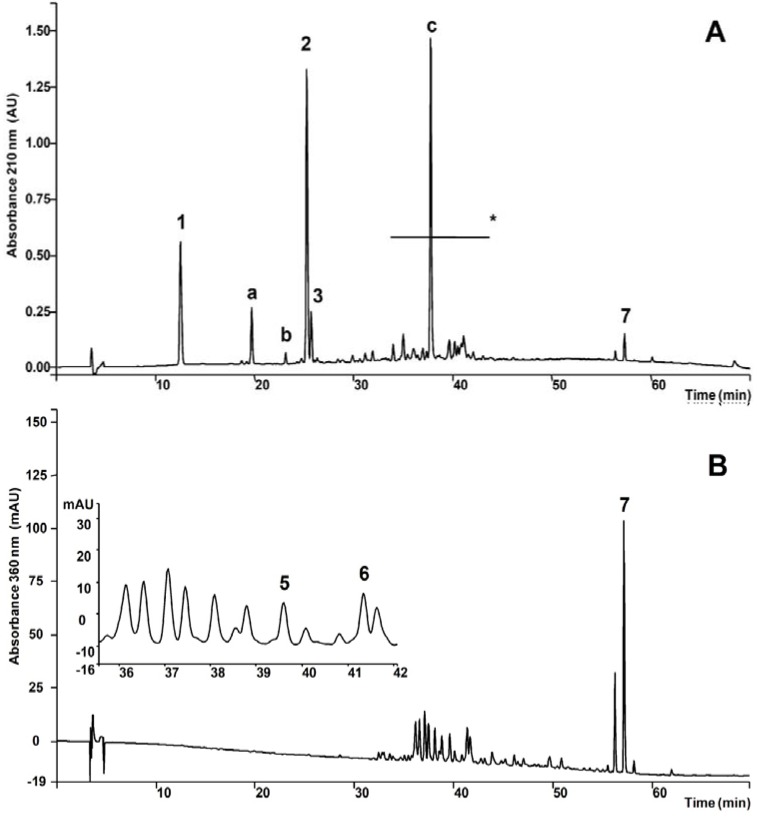
**A.** HPLC chromatographic profile of the AcF from *A. humile* monitored at 210 nm. 1. Gallic acid, a. Unknown, b. Unkown catechin, 2. Methyl gallate, 3. (+)-catechin, c. Gallic acid derivative, 7. Amenthoflavone, * Flavonoid glycosides and gallic acid derivatives. **B.** Elution monitored at 360 nm for flavonol glycosides.

The chromatographic profile of *A. humile* presented classes of compounds (Figure 1 and Figure 2 and Table 1) which have beneficial effects on gastrointestinal ulcers [18], justifying the assessment of antiulcer activity and the mechanisms involved.

Examination of the chromatograms shown in Figure 2A led to the recognition of three classes of secondary metabolites in AcF (Table 1): gallic acid derivatives, catechins and flavonoids (flavonoid glysosides and biflavonoid). Flavonoid glycosides were better detected at 360 nm (Figure 2B). Determination of each class was accomplished using HPLC analyses and external calibration.

**Table 1:** Concentration of the secondary metabolites present in the AcF from *A. humile* St. Hil.

Substance or class	Concentration ± SD (mg·g^-1^)
**Gallic acid derivatives**	
Gallic acid	103.08 ± 0.64
Methyl gallate	228.04 ± 1.11
Unknown gallic acid derivatives	329.59 ± 3.65
Total	728.71 ± 3.78
**Catechins**	
(+)-Catechin	21.14 ± 0.45
Unknown catechins	4.34 ± 0.08
Total	29.70 ± 0.46
**Flavonoids**	
Amenthoflavone	29.33 ± 0.82
Unknown flavonoids	92.76 ± 0.75
Total	122.09 ± 1.07

The effect of ethyl acetate extract from *Anacardium humile* (AcF), on gastric ulcers induced by an irritant agent (absolute ethanol) was investigated in rats. Pre-treatment with AcF and lansoprazole were found to inhibit ethanol-induced gastric mucosal injury. This inhibitory effect of AcF was the highest and statistically significant in the 50 mg·kg^−1 ^group. The groups treated with 100 mg·kg^−1^ and 200 mg·kg^−1^ of AcF, showed insignificant inhibitory effects for ethanol-induced gastric mucosal injury. Therefore, with the purpose of investigating the probable gastroprotective mechanisms involved in the action promoted by AcF, we studied only a dose of 50 mg.kg^−1 ^for its efficacy. At the same time, 30 mg·kg^−1^ of lansoprazole significantly inhibited ethanol-induced gastric lesions compared to the control. The obtained results suggested that the ethyl acetate extract of *A. humile* posses a significant antiulcer effect in these ulcer-induced models. 

**Table 2:** Effects of orally administered ethyl acetate extract (AcF; 50, 100 and 200 mg.kg^-1^) obtained from the leaves of *A. humile* St. Hil. on ethanol-induced gastric ulcers in rats.

Models	Groups	Ulcer index	Inhibition
	(n = 7)	(mean ± SD)	(%)
Ethanol	Vehicle	34.4 ± 14.2	-
	50 mg·kg^-1^ AcF	11.6 ± 6.6^a^	66.2
	100 mg·kg^-1^ AcF	22.8 ± 12.6	33.7
	200 mg·kg^-1^ AcF	31.4 ± 10.5	8.7
	30 mg·kg^-1^ lansoprazole	1.5 ± 0.8^a^	95.6

The columns are the mean ± S.D. of six rats. Different letters indicate significant differences. ANOVA: F_(4,22)_ = 8.521 (p < 0.05, Tukey test).

**Figure 3:**
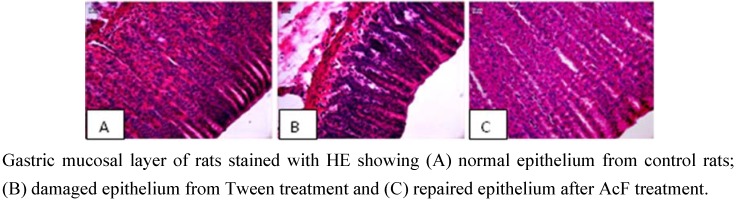
Photomicrography of stomach gastric ulceration caused by ethanol.

EtOH-induced ulcers were not inhibited by antisecretory agents such as cimetidine, but are inhibited by agents that exhibit a gastroprotective action with an anti-oxidative cytoprotection [19]. Results of the present study showed that AcF (50 mg·kg^−1^) provided protection against gastric ulcers induced by ethanol (Table 2 and Figure 3C). Ethanol-induced ulceration is inhibited by agents that enhance mucosal defensive factor [19,20]. Since AcF enhanced mucosal defensive factors (PGE_2_ and mucus), this increase of the defensive factors promoted by the AcF may be, at least partially, one of the possible mechanisms by which AcF ameliorated the ethanol-induced gastric damage. 

**Figure 4:**
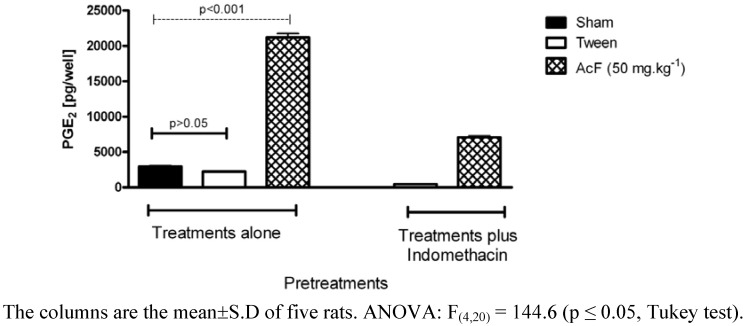
Effects of orally administered ethyl acetate extract (AcF; 50 mg·kg^−1^) obtained from the leaves of *A*. *Humile* St. Hil. and indomethacin on gastric prostaglandin E_2_ (PGE_2_) production in rats.

Aiming to investigate the probable gastroprotective mechanisms involved in the action promoted by this extract, we evaluated the role of AcF on PGE_2_ production. Pretreatment of rats with indomethacin markedly reduced the gastric mucosal prostaglandin contents (Figure 4). On the other hand, pretreatment of rats with AcF (50 mg·kg^−1^) induced drastic increase of PGE_2_ levels as compared to rats treated only with Tween (P < 0.01).

Continuous generation of PGE_2_ by the mucosa is crucial for the maintenance of mucosal integrity and protection against ulcerogenic and necrotizing agents [21]. Almost all of the mucosal defense mechanisms are stimulated and/or facilitated by PGs. Our data suggest that the cytoprotective action of AcF on the gastric mucosa may be related to an increase in PGE_2_ production. PGs inhibit acid secretion, stimulate mucus, bicarbonate, and phospholipid secretion; increase mucosal blood flow; and accelerate epithelial restitution and mucosal healing [21].

**Figure 5:**
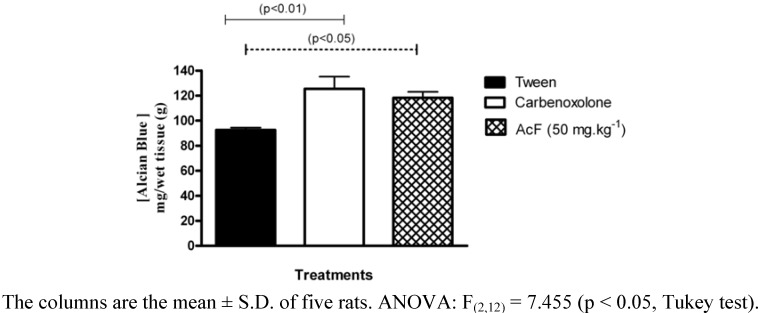
Effects of orally administered ethyl acetate extract (AcF; 50 mg·kg^−1^) obtained from the leaves of *A. humile* St. Hil. and of carbenoxolone (200 mg·kg^−1^) on adherent gastric mucous (measured as the amount of alcian blue bound) in pylorus-ligated rats.

We also observed the effect of AcF on adherent mucus production by the gastric mucosa (Figure 5). Pretreatment with AcF (50 mg.kg^−1^) and carbenoxolone (200 mg·kg^−1^) induced significant increase in mucoprotective effect in animals submitted to pylorus ligature.

**Figure 6:**
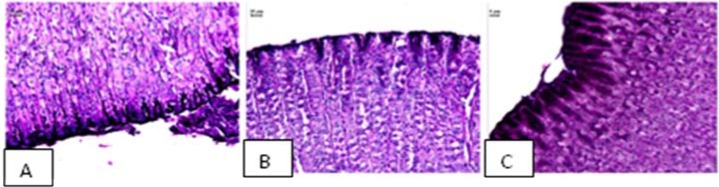
Photomicrography of stomach showing the mucosal layer stained with PAS showing mucus in (A) normal epithelium; (B) loss of epithelial mucus in Tween treatment and (C) presence of epithelial mucus after AcF treatment.

**Figure 7:**
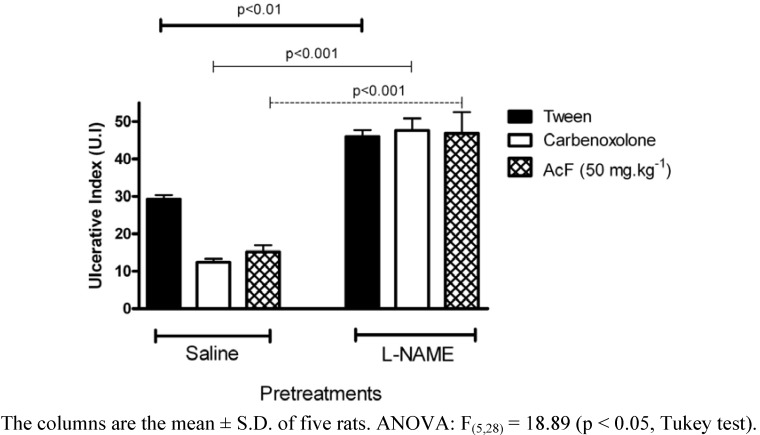
Effects of orally administered ethyl acetate extract (AcF; 50 mg·kg^−1^) obtained from the leaves of *A. humile* St. Hil. and of carbenoxolone (100 mg·kg^−1^) on ethanol-induced gastric ulcers in rats pretreated with L-NAME.

Gastric mucus is an important protective factor for the gastric mucosa and consists of a viscous, elastic, adherent and transparent gel formed by water and glycoproteins that covers the entire gastrointestinal mucosa. The protective properties of the mucus barrier depend not only on the gel structure but also on the amount or thickness of the layer covering the mucosal surface [22]. In the present study, we also measured gastric adherent mucus and, the production was increased in AcF (50 mg·kg^−1^) (Figure 5 and Figure 6C). Furthermore, our findings that AcF stimulated mucus secretion support the notion that this fraction acted by stimulating PGE_2_ production (Figure 7C). It has been previously reported that PGE_2_ stimulates mucus secretion [22]. The mucus layer protects the newly formed cells against damage caused by acid pH and the proteolytic potential of the gastric secretions [23].

Vascular changes in gastric mucosa appeared to be the most pronounced feature of absolute ethanol-induced injury [24]. As shown in Figure 7, pretreatment with NG-nitro-L-arginine methyl ester (L-NAME) (70 mg·kg^−1^, i.p.) attenuated the gastroprotection of both AcF (50 mg·kg^−1^) and carbenoxolone (100 mg·kg^−1^). 

Nitric oxide (NO) synthesis or exogenously given NO has been repeatedly shown to protect the gastric mucosa against damage induced by various agents [25]. It has been reported that the serum and local NO levels are reduced in gastric injury models induced by ethanol and indomethacin, suggesting that the decrease in local NO content might be a key factor in facilitating gastric mucosal injury [26]. The previous administration of L-NAME, an NO-synthase inhibitor, altered the cytoprotection induced by AcF, suggesting that the anti-ulcer activity of this fraction is through the participation of endogenous nitric oxide. The role of NO in gastroprotection has been widely accepted [27]. An increase in NO levels by L-arginine (a substrate for NOS), but not D-arginine, has been shown to reduce absolute ethanol- induced gastric lesions [28].

**Figure 8:**
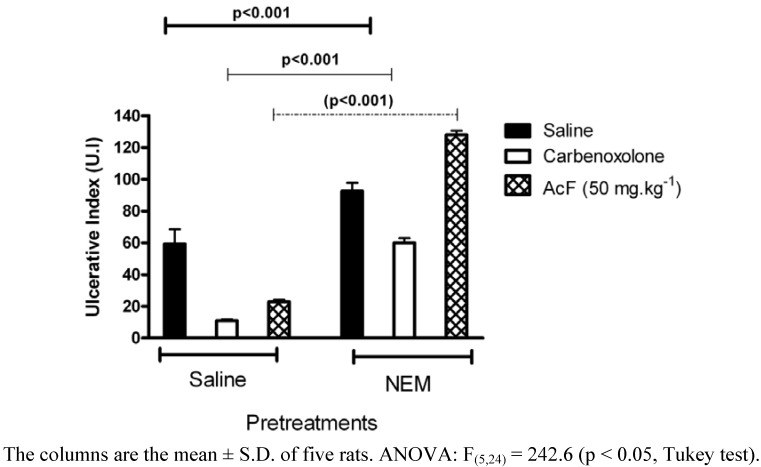
Effects of orally administered ethyl acetate extract (AcF; 50 mg·kg^−1^) obtained from the leaves of *A. humile* St. Hil. and of carbenoxolone (100 mg·kg^−1^) on ethanol-induced gastric ulcers in rats pretreated with NEM.

It is well known that reduced glutathione GSH protects the gastric mucosa submitted to an ulcerative challenge [29]. *N*-Ethylmaleimide (NEM, 10 mg·kg^−1^, s.c.)-pretreated rats produced a reduction in the gastroprotection exerted by the administration of AcF 50 mg·kg^-1^ on ethanol-induced gastric hemorrhagic lesions (Figure 8). A significant decrease in gastric GSH following ethanol administration indicated massive generation of free radicals [30]. Our result is in agreement with earlier reports showing depletion of sulfhydryls in ethanol-induced gastric lesions [31]. In this respect, the present increase of tissue damage, evoked by ethanol in the NEM pretreated rats, was expected because the treatment with glutathione depletors has been shown to significantly potentiate ulcerogen-induced gastric mucosal injury [32], whereas an increase in mucosal NP-SH exerts a gastroprotective effect [33]. In this context, the antiulcerogenic activity of AcF (50 mg·kg^−1^) may depend on mucosal GSH levels, and it is likely that an increase of endogenous sulfhydryl compounds play an important role in the gastroprotective properties of this fraction. 

## Experimental

### Plant material, extraction and isolation

*A. humile* St. Hil. was collected along Monte do Carmo road, in Porto Nacional in Tocantins state, Brazil in November 2002. The plants were identified by Marcos Alves and Eduardo Ribeiro of the University of Tocantins and a voucher specimen (accession number 1922) was deposited in the University of Tocantins Herbarium.

Air-dried and powdered leaves (650 g) of *A. humile* St. Hil. were exhaustively extracted by successive maceration at room temperature with dichloromethane (DCM, 5 L) and methanol (MeOH, 5 L) (130:1, w/v, one week for each solvent). Solvents were evaporated at 60 °C under reduced pressure to yield the DCM (6 g) and MeOH (193 g) extracts. A portion of the MeOH extract (80 g) was partitioned between a mixture of EtOAc/water (5 L, 1:1, v/v) to yield 3.4 g of EtOAc fraction (AcF) and 74 g of aqueous fraction (AqF). Flavonoids were detected in AcF as described elsewhere [34].

A portion (1 g) of the MeOH extract of *A. humile* St. Hil. was fractionated by gel permeation chromatography on a Sephadex LH-20 (Pharmacia) column (5 cm × 100 cm) and eluted with MeOH (flow rate 0.5 mL·min^−1^). One hundred fractions (5 mL each) were collected and checked by Thin Layer Chromatography (TLC) on silica gel plates eluted with a mixture of CHCl_3_/MeOH/n-PrOH/H_2_O (5:6:1:4; lower phase) and developed either with Natural Products - Polyethyleneglycol Reagent or with anisaldehyde/sulfuric acid solution [34]. The fractions were subsequently combined and then purified by HPLC using a Knauer Chance system equipped with a Waters R401 refractive index detector, a Phenomenex Luna reverse-phase RP 18 column (25 cm × 1 cm × 10 μm) and a Rheodyne injector with a 100 μl sample loop. MeOH-H_2_O (8:2, v/v) was used as the eluent (flow rate 2 mL·min^‑1^). Fractions 64-72 (150 mg) yielded gallic acid (**1**, 50 mg) and methyl gallate (**2**, 75 mg); fractions 98-103 (75 mg) yielded (+)-catechin (**3**, 50 mg); fractions 119-124 (35 mg) yielded quercetin-3-*O*-*β*-D-allopyranoside (**4**, 5 mg) and quercetin-3-*O*-*β*-D-galactopyranoside (**5**, 8 mg); fractions 144–149 (35 mg) yielded quercetin-3-*O*-*α*-L-rhamnopyranosyl-(1→6)*-β*-D-glucopyranoside (rutin, **6**, 8 mg); and fractions 178–200 (200 mg) yielded the biflavonoid amentoflavone (**7**, 165 mg) (Figure 1). 

The chemical identification of substances (**1–7**) was established by Nuclear magnetic resonance Nuclear magnetic resonance (NMR) spectra in DMSO-d_6 _using a Varian INOVA 500 spectrometer operated at 500 MHz for ^1^H and 150 MHz for ^13^C. The 2D analyses included ^1^H-^1^H-COSY (chemical shift correlation spectroscopy), inverse-detected ^1^H-^13^C HSQC (heteronuclear single quantum coherence) and HMBC (heteronuclear multiple bond connectivity). All of the spectral data of the substances (**1-7**) were compared to those reported in the literature [35,36].

The chromatographic profile of AcF was obtained using an HPLC system (ProStar, Varian) equipped with a ProStar 330 photodiode-array ultraviolet detector (PDA), a Rheodyne injector (20 µL), a Phenomenex Luna RP-18 column (250 × 4.6 mm i.d. 5 μm) and a RP-18 Phenomenex guard column (4 × 4.6 mm, i.d. 5 µm). Elution was performed with a linear gradient of water (A) and acetonitrile (B) (with 0.05% of trifluoroacetic acid) from 23 to 30% of B in 5 min, then from 30 to 48% of B in 40 min and then from 48 to 100% of B in 65 min. Flow rate was 1.0 mL.min^−1^ and effluent was monitored at 210 nm and at 360 nm. 

The determination of the concentration of the metabolites present in AcF was performed using external calibration. Flavonoids were expressed based on rutin, in the calibration range between 5–500 µg·mL^–1^ (y = -4.81·10^5^ + 1.91·10^5 ^x, R = 0.9999, N = 6); gallic acid derivatives were expressed based on gallic acid, in the calibration range between 2–200 µg·mL^–1^ (y = -9.69·10^4^ + 2.38·10^5 ^×, R = 0.9998, N = 9); catechins were expressed in terms of (+)-catechin, in the calibration range between 1–70 µg·mL^–1^ (y = 1.40·10^5^ + 8.93·10^5 ^x, R = 0.9999, N = 6).

### Animals

Male Wistar rats (180–250 g) obtained from the breeding colony at the Universidade Estadual de Campinas (CEMIB/UNICAMP) were used. The animals were housed in a 12 h light/dark cycle, at a humidity of 60 ± 10% and a temperature of 21.5 ± 1.0 °C and were fed a certified Nuvilab CR-a^℘ ^(Nuvital) diet, with free access to tap water. All experiments were carried out in the morning. The experimental protocols were all approved of by the Institutional Committee for Ethics in Animal Experimentation (no. 538-1, CEEA/IB/UNICAMP). 

### Drugs

Lansoprazole (Medley, Campinas, SP, Brazil), carbenoxolone and Tween 80^℘^ (Sigma Chemical Co., St. Louis, MO, USA.) were used in this study. The reagents for buffers and other solutions were all of analytical grade. All the other chemicals and reagents used in this study were of analytical grade.

### Ethanol-induced gastric lesion in rats

After a total of 24 h fasting, three groups of rats (n = 5) received an oral administration of AcF (50, 100, 200 mg·kg^−1^), lansoprazole (30 mg·kg^−1^) or vehicle (10 mL·kg^−1^). One hour after treatment, all rats received, orally, 1 mL of 99.5% ethanol to induce gastric ulcers [20]. The animals were killed by CO_2_ gas 1 h after treatment with the ulcerogenic agent and the stomachs removed to determine the gastric damage. The stomachs were removed, opened along the greater curvature and fixed between two glass plates. Ulcerative lesion was calculated according to the methodology described by Szelenyi and Thiemer [37].

### Determination of prostaglandin PGE2 synthesis

Thirty minutes after treatment with indomethacin (30 mg·kg^−1^), vehicle (10 mL·kg^−1^) or AcF (50 mg·kg^−1^), the rats were killed by CO_2_ gas and their abdomen opened. The control group without treatment experienced the same general conditions of the experimental groups. Samples of the corpus (full thickness) were excised, weighed and suspended in 1 mL of 10 mM sodium phosphate buffer, pH 7.4. After homogenizing with a Polytron^® ^PT 10-35 homogenizer (Kinematica AG, Lucerne, Switzerland), the homogenate was incubated in a Dubnoff water-bath (Tecnal, Piracicaba, Brazil) at 37 °C for 20 min and the amount of PGE_2_ in the buffer was measured by enzyme immunoassay using a commercial kit (RPN222, Amersham). The absorbance was read at 450 nm and the amount of PGE_2_ expressed as pg/wet weight of tissue, was determined from a standard curve of PGE_2 _[38].

### Determination of mucus in gastric content

After rats (n = 6–7) had fasted for 24 h, under anesthesia, the abdomen was incised and the pylorus ligated. The vehicle (Tween), carbenoxolone (200 mg·kg^−1^) or AcF (50 mg·kg^−1^) was administered orally after the pylorus ligature. The animals were killed by CO_2_ gas 4 h after the drug treatments. The stomach content was immersed in 10 mL 0.02% Alcian blue in 0.16 M sucrose/0.05 M sodium acetate, pH 5.8, and incubated for 24 h at 20 °C. The Alcian blue binding extract was centrifuged at 2000 × g for 10 min. The absorbance of supernatant was measured at 615 nm using a light spectrophotometer U/2000 (Hitachi, Japan). The free mucus in the gastric content was calculated from the amount of Alcian blue binding [mg/wt tissue (g)] [39].

### Ethanol-induced gastric lesion in NEM and L-NAME- pretreatment rats

Rats were divided into groups of 6–7 animals that fasted for 24 h. They had previously been treated intraperitoneally with NEM (*N*-ethylmaleimide, Sigma, USA) at a dose of 10 mg·kg^−1^, L-NAME (NG-nitro-L-arginine methyl ester, Sigma, USA) at a dose of 70 mg·kg^−1^ or saline. Thirty minutes later, the groups received an oral dose of the vehicle, carbenoxolone (100 mg·kg^−1^) or AcF (50 mg·kg^−1^). After 60 min, all groups were treated orally with 1 mL of absolute ethanol for gastric-ulcer induction [40]. Animals were killed by CO_2_ gas 1 h after ethanol administration and the stomachs excised and gastric damage determined as described above.

### Histology of ethanol-induced gastric lesions

The stomach of the rats submitted to gastric ulcers in the ethanol model with different treatments (control, carbenoxolone and AcF) were pushed off and opened by the large curvature and the lesion was localized. The lesion was sectioned, and one sample was fixed in ALFAC solution (alcohol, chloroform and acetic acid) for 24 h in 4 °C. The samples were routinely processed for embedding in paraplast, and cut into 7 µm thick section. These sections were stained with hematoxylin-eosin [41] and periodic acid –Schiff (PAS) [42]. The samples were analysed with a Leica microscope associated with Leica Qwin Software (Leica-England). 

### Statistical analysis

The results were expressed as the mean ± S.D. Statistical significance among groups was assessed by one-way analysis of variance (ANOVA) followed by the Tukey tests, with p ≤ 0.05 indicating significance. All statistical analyses were done using Prism software (GraphPad, San Diego, CA, USA).

## Conclusions

In conclusion, only a limited number of studies have reported the biological effects of *Anacardium humile* St. Hil., which has made it difficult to find references, including those describing anti-ulcerogenic activity, first reported by our research group in a previous study. The present investigation attempted to evaluate in more depth the anti-ulcerogenic mechanism of the ethyl acetate extract of this plant. The gastroprotective mechanism is based on its ability to strengthen defensive factors by elevating mucus and PGE_2_ levels with participation of NO and SH groups to prevent or attenuate the ulcer process. Therefore, the results obtained from the administration of AcF of *Anacardium humile* under *in vivo* models support the ethnopharmacological use of this species.
